# A translational approach to test anti-inflammatory drugs in a LPS induced acute lung inflammation model in the common marmoset (*Callithrix jacchus*)

**DOI:** 10.1186/1476-9255-10-S1-P28

**Published:** 2013-08-14

**Authors:** S Seehase, S Switalla, V Neuhaus, M Zöller, FJ Kaup, C Schlumbohm, E Fuchs, HD Lauenstein, K Sewald, JM Hohlfeld, A Braun, S Knauf

**Affiliations:** 1 Fraunhofer ITEM, Hannover, Germany; 2 German Primate Center, Leibniz-Institute for Primate Research, Göttingen, Germany; 3 Encepharm GmbH, Göttingen, Germany

## 

The rising incidence of chronic-obstructive pulmonary diseases is linked to the development of new human-specific anti-inflammatory therapeutics. In many cases classic rodent models fail to predict reliable data of safety and efficacy, which contributes to a growing demand of models that represent the human situation in more detail e. g., anatomy and immune-response.

Similar to the human setting of segmental lipopolysaccharide (LPS) challenge, we established a LPS induced acute lung-inflammation model in the common marmoset monkey, a small non-human primate. LPS induced inflammation mimics pro-inflammatory aspects of chronic airway diseases associated with neutrophile aggregation.

Healthy animals underwent a first BAL to obtain baseline data. Marmosets were than pre-treated orally with roflumilast (7 µg/kg bw), a recently approved PDE-4-inhibitor, or dexamethasone (2 mg/kg bw) as a treatment control on five consecutive days. Sham treated animals served as positive control. Under general anaesthesia 500 ng LPS were instilled into the left half of the lung of each animal, followed by ipsilateral BAL of the LPS challenged lung 18 h post-provocation. BAL fluid was processed and analysed for cellular and cytokine levels. A whole blood assay performed on day 0 and 5 served as an additional readout.

As expected, LPS induced significant influx of total cells into the airways (p<0.001) mainly through neutrophil aggregation. Pre-treatment with roflumilast resulted in a statistically significant decrease of neutrophil numbers in BAL fluid (p=0.047). Dexamethasone pre-treatment showed a significant reduction in relative cell numbers (p=0.047), but not in absolute neutrophil numbers (p=0.076). TNF-α levels were significantly increased after LPS provocation and significantly suppressed in roflumilast (p=0.048) and dexamethasone (p=0.036) pre-treated animals. In the WBA only pre-treatment with dexamethasone resulted in a reduced TNF-α release after *ex vivo* LPS provocation.

Results from this new translational nonhuman primate model indicate the common marmoset monkey as a promising species to test new anti-inflammatory drugs.

